# Hydroxychloroquine withdrawal triggers pregnancy-associated pulmonary arterial hypertension in systemic lupus erythematosus: a case report and exploration of the Complement-EndMT axis

**DOI:** 10.3389/fphar.2026.1778983

**Published:** 2026-03-02

**Authors:** Yu-Fei Zhang, Chun-Fei Wang, Li Zhang, Xue-Feng Jiao, Jin-ke Li, Qiang Wei

**Affiliations:** 1 Department of Obstetrics and Gynecology, West China Second University Hospital, Sichuan University, Chengdu, China; 2 Key Laboratory of Birth Defects and Related Diseases of Women and Children, Sichuan University, Ministry of Education, Chengdu, China; 3 Department of Pharmacy/Evidence-Based Pharmacy Center, West China Second University Hospital, Sichuan University, Children's Medicine Key Laboratory of Sichuan Province, Chengdu, China; 4 NMPA Key Laboratory for Technical Research on Drug Products In Vitro and In Vivo Correlation, Chengdu, China

**Keywords:** complement system, drug adherence, endothelial-mesenchymal transition, hydroxychloroquine, pregnancy, pulmonary arterial hypertension, systemic lupus erythematosus

## Abstract

**Background:**

The continuation of hydroxychloroquine (HCQ) during pregnancy in patients with systemic lupus erythematosus (SLE) is a cornerstone of management, proven to mitigate maternal disease flares. However, its precise role in preventing the devastating cardiopulmonary complication of pregnancy-associated pulmonary arterial hypertension (PAH) remains inadequately defined, and the underlying pharmacological mechanisms remain largely elusive.

**Case presentation:**

We detail the case of a 31-year-old primigravida with a 15-year history of well-controlled SLE, who self-discontinued HCQ at 8 weeks of gestation. At 27 + 4 weeks, she presented with significant exertional dyspnea. Diagnostic evaluation confirmed severe PAH (estimated PASP 107 mmHg) with right heart strain, alongside serological evidence of active SLE, including hypocomplementemia. A multidisciplinary therapeutic protocol was immediately instituted, comprising the reinstatement of HCQ and the administration of intravenous methylprednisolone. This intervention resulted in a marked reduction in pulmonary arterial pressure to a moderate range (PASP 73 mmHg), stabilizing the patient’s condition sufficiently to prolong gestation to 31 + 1 week, culminating in a planned cesarean delivery. At the 3-month postpartum assessment, echocardiography documented sustained improvement, with PAH decreased to a mild grade (PASP 40 mmHg).

**Conclusion:**

This case provides compelling *in vivo* evidence that non-adherence to HCQ constitutes a pivotal, modifiable risk factor for the onset of SLE-associated PAH in the gravid state, and that pharmacological reintroduction can arrest and partially reverse this pathogenic trajectory. We attribute the vascular protective effects of HCQ to the inhibition of complement activation along the C5a-MAPK/ERK signaling axis. Targeting this pathway disrupts pathological endothelial-mesenchymal transition (EndMT) and mitigates subsequent pulmonary vascular remodeling. Stringent HCQ adherence should be standard of care. Furthermore, complement monitoring guides precision pharmacotherapy to prevent PAH in susceptible SLE pregnancies.

## 1 Introduction

Systemic Lupus Erythematosus (SLE) is a prototypical systemic autoimmune disorder characterized by multisystem involvement and pathogenic autoantibody production (Silver et al., 2023; Humbert et al., 2023). In the general population, the global incidence of SLE is estimated to be 5.14 (1.4–15.13) per 100,000 person-years, representing approximately 0.40 million new cases annually. Incidence rates vary significantly geographically; for instance, in China, the estimated incidence is 8.57 (8.37–8.77) per 100,000 person-years (Tian et al., 2023). Besides, this disorder exhibits a pronounced predilection for women of reproductive age, with a female-to-male ratio of approximately 9:1. Consequently, the management of SLE during pregnancy presents a formidable clinical challenge at the intersection of rheumatology, obstetrics, and cardiology. Pregnancy induces a unique immunological and endocrinological milieu characterized by a shift towards T-helper 2 (Th2) dominance, elevated estrogen and progesterone levels, and altered complement regulation. For women with SLE, these physiological adaptations can disrupt immune homeostasis, thereby precipitating disease flares (Silver et al., 2023; Humbert et al., 2023). Active disease is clinically significant as it is strongly associated with a spectrum of severe adverse pregnancy outcomes, including preeclampsia, fetal growth restriction, spontaneous preterm birth, and increased perinatal mortality (Wind et al., 2024; Sun et al., 2025; Singh et al., 2023).

Within the spectrum of severe cardiopulmonary complications associated with SLE, pulmonary arterial hypertension (PAH) is distinguished by its insidious onset and dire prognosis. SLE-associated PAH (SLE-PAH) is recognized as a distinct subgroup within Group 1 pulmonary hypertension according to contemporary classification schemes (Humbert et al., 2023). Its pathogenesis is paradigmatic of a multifactorial, synergistic insult to the pulmonary vasculature. Key contributors include the deposition of circulating immune complexes within vessel walls, dysregulated activation and consumption of the complement cascade, sustained production of pro-inflammatory cytokines (e.g., IL-6, TNF-α), and direct endothelial injury mediated by a repertoire of autoantibodies, such as anti-endothelial cell antibodies (AECAs) and anti-U1 ribonucleoprotein (RNP) antibodies (Zhang et al., 2024). These convergent pathological insults initiate and perpetuate a vicious cycle of endothelial dysfunction, hyperproliferation of pulmonary arterial smooth muscle cells, perivascular inflammation, and *in situ* thrombosis. The histological endpoint represents the hallmark of PAH: progressive, obstructive pulmonary vascular remodeling, culminating in elevated pulmonary vascular resistance, right ventricular pressure overload, and eventual right heart failure with a historically high associated mortality (Ranchoux et al., 2015; Singh et al., 2024).

Hydroxychloroquine (HCQ), a 4-aminoquinoline derivative, has evolved from an antimalarial agent to an indispensable cornerstone of SLE pharmacotherapy. Its pleiotropic benefits are well-established, and international consensus guidelines uniformly advocate for its uninterrupted continuation throughout pregnancy (Sammaritano et al., 2020). The evidence base supporting this recommendation is robust, demonstrating HCQ’s efficacy in reducing overall disease activity, preventing clinical flares, diminishing the risk of thrombosis (particularly in the context of antiphospholipid antibodies), and potentially improving long-term survival and neonatal outcomes (Liu et al., 2024). The drug’s primary pharmacological mechanisms are attributed to its immunomodulatory properties, which include raising endosomal pH to inhibit Toll-like receptor (TLR) 7 and 9 signaling, a key pathway for type I interferon production in SLE, and modulating aberrant T-cell activation and cytokine profiles (Rainsford et al., 2015).

However, while systemic immunomodulatory efficacy of HCQ is well-established, its potential for direct, organ-specific vascular protection remains mechanistically elusive. Specifically, whether HCQ engages the discrete pathobiological cascades driving pulmonary vascular remodeling, effects that transcend generalized immunosuppression, represents a critical knowledge gap. This deficit has tangible clinical repercussions: it may lead to an underemphasis on the vital importance of HCQ adherence during prenatal counseling, thereby inadvertently exposing patients to preventable, life-threatening cardiopulmonary sequelae.

This manuscript presents a compelling case report that exemplifies the critical consequences of HCQ withdrawal during pregnancy and the therapeutic reversibility achieved upon its reintroduction. We leverage this clinical narrative as a foundation to synthesize extant literature and propose a novel, integrative mechanistic hypothesis. Specifically, we postulate that HCQ transcends its role as a broad-spectrum immunomodulator, potentially acting as a vital preserver of pulmonary vascular integrity. This is achieved through the targeted suppression of the complement-driven endothelial-mesenchymal transition (EndMT) axis. By bridging the critical gap between clinical observation and molecular pathogenesis, this exploration offers fresh insights for both patient management and future investigative directions.

## 2 Case presentation

### 2.1 Patient information and clinical history

A 31-year-old primigravida was urgently referred to our tertiary academic medical center at a gestational age of 27 weeks and 4 days. She reported worsening shortness of breath, which had made it difficult for her to walk more than a few meters on flat ground. This was classified as New York Heart Association (NYHA) functional class III. Her medical history included SLE, diagnosed 15 years ago based on a malar rash, joint inflammation, positive antinuclear antibodies (ANA), and high anti-double-stranded DNA (anti-dsDNA) antibodies. For over a year prior to conception, she had maintained stable clinical and serological remission on hydroxychloroquine (200 mg twice daily). No other immunosuppressive agents were required. Following confirmation of pregnancy, the patient discontinued HCQ therapy at approximately 8 weeks of gestation, motivated by concerns regarding potential teratogenicity. This critical decision was made without prior consultation with her rheumatologist or obstetrician.

### 2.2 Diagnostic assessment

Upon admission, the patient appeared anxious and shortness of breath at rest. Vital signs were notable for tachycardia (heart rate 108 beats per minute) and tachypnea (respiratory rate fluctuating between 17 and 32 breaths per minute). Blood pressure was 125/80 mmHg, and oxygen saturation was 94% on room air. Cardiovascular examination revealed a prominent, palpable P2 component at the pulmonic area and a loud, high-pitched second heart sound upon auscultation. A grade 3/6 holosystolic murmur, best audible at the left lower sternal border and increasing with inspiration, was detected, consistent with significant tricuspid regurgitation. There was no peripheral edema, cyanosis, or clubbing. Pulmonary auscultation was unremarkable. No active cutaneous or articular stigmata of SLE were present.

Laboratory Investigations: Comprehensive serological profiling was performed. Results confirmed active SLE serology: ANA was positive at a titer of 1:320 with a speckled pattern, and anti-dsDNA antibodies were elevated. Critically, there was biochemical evidence of complement consumption, with significantly depressed serum levels of C3 (0.65 g/L; reference range 0.9–1.8 g/L) and C4 (0.10 g/L; reference range 0.1–0.4 g/L). Routine hematological parameters, renal function tests (serum creatinine, estimated glomerular filtration rate), and liver function panels were all within normal limits, arguing against concurrent active lupus nephritis or hepatitis.

Echocardiography: Transthoracic echocardiography was the cornerstone of diagnosis. It revealed profound right heart dilation, including severe enlargement of the right atrium and right ventricle. The interventricular septum exhibited paradoxical flattening during systole, indicative of right ventricular pressure overload. Continuous-wave Doppler interrogation across the tricuspid valve demonstrated severe regurgitation, with a maximum regurgitant jet velocity of approximately 5.1 m/sec. Applying the modified Bernoulli equation, this translated to an estimated right ventricular systolic pressure (RVSP), equated to pulmonary arterial systolic pressure (PASP) in the absence of pulmonary stenosis, of 107 mmHg, diagnostic of severe PAH. The left ventricular cavity size was normal, and systolic function was preserved with an ejection fraction of 74%. No significant left-sided valvular disease or pericardial effusion was noted.

Obstetric Ultrasound: A detailed fetal anatomical survey showed a singleton fetus with biometry consistent with the reported gestational age. Amniotic fluid volume was normal, and umbilical artery Doppler studies were reassuring, with no evidence of fetal growth restriction or hemodynamic compromise at that juncture.

A synthesis of the clinical presentation, serological activity, and profound echocardiographic findings led to the definitive diagnosis of severe, pregnancy-associated SLE-PAH.

### 2.3 Therapeutic intervention and pharmacological management

Recognizing the acute, life-threatening nature of the condition, an urgent multidisciplinary team (MDT) conference was convened within hours of admission. The assembly included specialists from rheumatology, obstetrics, cardiology, pulmonology, anesthesiology, and neonatology. The consensus therapeutic strategy was twofold, aiming to simultaneously suppress the underlying autoimmune drive and mitigate pulmonary vascular pressure.

Reinitiation of Immunomodulatory Therapy: Oral hydroxychloroquine was immediately recommenced at the patient’s prior dose of 200 mg twice daily, targeting the root SLE activity.

Initiation of Anti-inflammatory Therapy: Given the severity of the presentation and serological activity, intravenous methylprednisolone was administered at a dose of 40 mg daily for 3 days to achieve rapid cytoreduction and inflammatory control, after which it was transitioned to an equivalent oral dose of prednisone.

Adjunctive Thromboprophylaxis: Considering the hypercoagulable state of both pregnancy and SLE, prophylactic-dose low-molecular-weight heparin (enoxaparin) was initiated.

The clinical response was closely monitored. Within 72 h, the patient reported a subjective improvement in her dyspnea. A follow-up echocardiogram performed 2 weeks after treatment initiation documented a substantial and encouraging reduction in the estimated PASP from 107 mmHg to 73 mmHg, corroborating the clinical amelioration. The right heart dimensions, while still enlarged, showed early signs of improvement.

### 2.4 Follow-up and outcomes

Despite the favorable initial response, the patient’s PAH remained in the moderate range (PASP ∼73 mmHg), and the inherent hemodynamic stresses of advanced pregnancy posed a continuing, unacceptable risk for maternal decompensation or sudden death. After extensive and candid discussions with the patient and her family regarding the profound risks of continuing the pregnancy versus the prematurity-related risks to the neonate, a joint decision was made for planned delivery. At 31 weeks and 1 day of gestation, under carefully titrated combined spinal-epidural anesthesia to avoid systemic vasodilation and cardiovascular collapse, a lower-segment cesarean section was successfully performed. A viable female infant weighing 1760 g was delivered with excellent Apgar scores of 10, 10, and 10 at 1, 5, and 10 min, respectively. The neonate was transferred to the neonatal intensive care unit for anticipated preterm care, and the maternal intraoperative and immediate postpartum course was remarkably uneventful.

**FIGURE 1:**
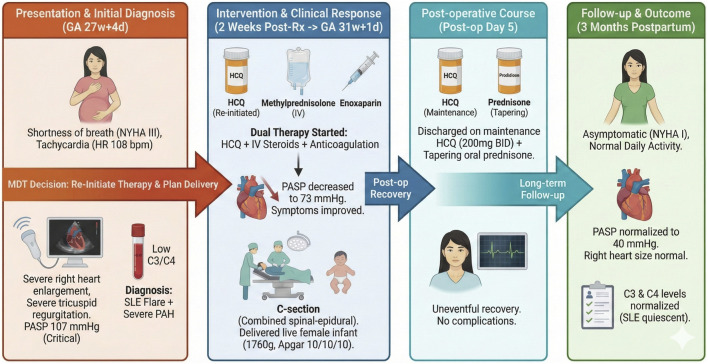
Clinical timeline of pregnancy-associated SLE-PAH triggered by HCQ withdrawal and resolution following therapy Re-initiation.

The patient was discharged home on postoperative day 5 on her maintenance HCQ (200 mg BID) and a tapering schedule of oral prednisone. At her comprehensive 3-month postpartum follow-up visit, she was entirely asymptomatic, engaging in all normal daily activities without limitation (NYHA class I). Repeat echocardiography revealed a heartening normalization of right heart chamber sizes and continued decline in pulmonary pressures. The estimated PASP had fallen to 40 mmHg, consistent with only mild residual PAH. Concurrently, serological reassessment showed complete normalization of her complement C3 and C4 levels, indicating effective suppression of her SLE disease activity. A timeline of the case report is shown in Figure 1.

## 3 Discussion

Clinical guidelines, such as those from EULAR and ACR, recommend hydroxychloroquine (HCQ) for all SLE patients unless contraindicated, typically at a target dose of 5 mg/kg real body weight/day, with individualization based on the risk of flares and retinal toxicity (Fanouriakis et al., 2023). The documented clinical trajectory carries profound mechanistic implications. The abrupt withdrawal of HCQ precipitated severe PAH, while subsequent drug reintroduction induced rapid hemodynamic recovery. This distinct temporal relationship strongly substantiates a causal link. This sequence powerfully elevates HCQ from a background maintenance agent to an active, non-redundant player in pulmonary vascular protection in the vulnerable context of SLE pregnancy. The partial but significant reversibility of the PAH process upon treatment reinstatement is a critical observation. While the concomitant use of corticosteroids likely mitigated the acute inflammatory surge, the sustained hemodynamic improvement observed postpartum under a regimen anchored by HCQ strongly suggests a specific and durable therapeutic effect. This likely reflects the targeting of pathogenic pathways driving vascular dysfunction and remodeling, extending efficacy beyond transient immunosuppression.

This clinical observation aligns with and is corroborated by a growing body of literature regarding the pleiotropic, vascular-protective pharmacology of HCQ. These multifaceted effects include the improvement of endothelial function via modulation of nitric oxide synthase (eNOS) activity and reduction in oxidative stress (Rainsford et al., 2015); the provision of antithrombotic properties through inhibition of platelet aggregation and antagonism of antiphospholipid antibody effects (Rainsford et al., 2015); and the modulation of critical cellular processes such as autophagy—a dysregulated pathway implicated in the hyperproliferative and apoptosis-resistant phenotype of pulmonary vascular cells in PAH (Ranchoux et al., 2015; Singh et al., 2024). Our case provides clinical validation for the premise that HCQ functions as a dual-action agent, serving as both an ‘immunological stabilizer’ and a ‘vascular remodeling modulator.'

### 3.1 Physiological stress and multiorgan involvement in maternal SLE-PAH

The pathophysiology of PAH in SLE is multifactorial, involving chronic inflammation, immune complex deposition, and progressive vascular remodeling. Autoantibodies, particularly anti-U1RNP and antiphospholipid antibodies, trigger endothelial activation and dysfunction, causing an imbalance between vasoconstrictors (e.g., endothelin-1) and vasodilators (e.g., nitric oxide) (Dhala, 2012; Humbert et al., 2023). Pregnancy exacerbates these underlying mechanisms through distinct physiological adaptations. Physiological hypervolemia and increased cardiac output impose a significant hemodynamic burden on the already compromised pulmonary vascular bed. Furthermore, the hyperestrogenic milieu of pregnancy modulates immune responses, potentially triggering SLE flares that accelerate pulmonary vasculitis and *in situ* thrombosis. Unlike non-pregnant individuals, pregnant patients with SLE-PAH often present with rapidly progressive right heart failure, particularly in the late second trimester or early postpartum period, when cardiovascular reserves fail to compensate for elevated pulmonary vascular resistance (Dhala, 2012; Humbert et al., 2023).

Given that SLE is a systemic autoimmune disease, extra-pulmonary involvement frequently complicates the clinical management of PAH (Dhala, 2012). Concomitant cardiac manifestations, such as pericarditis, myocarditis, or valvular disease (e.g., Libman-Sacks endocarditis), can exacerbate hemodynamic instability (Dhala, 2012). While PAH remained the primary concern in this case, the systemic nature of SLE necessitated vigilance regarding lupus nephritis and neuropsychiatric SLE (Humbert et al., 2023). Renal impairment, characterized by proteinuria and hypertension, predisposes patients to fluid overload, thereby aggravating right ventricular preload (Humbert et al., 2023). Similarly, neuropsychiatric manifestations driven by the central nervous system vasculitis or autoantibodies may be unmasked or exacerbated during pregnancy-related flares (Dhala, 2012). Ultimately, multidisciplinary evaluation of these systemic involvements is crucial, as multisystem failure remains a leading driver of maternal mortality in severe SLE flares (Singh et al., 2023).

### 3.2 Proposed pharmacological mechanism: targeting the complement-EndMT axis

The systemic inflammatory milieu in SLE, characterized by a dysregulated cytokine network, serves as the initiating driver for pulmonary vascular remodeling (Dhala, 2012). Cytokines play a pivotal role in orchestrating both systemic SLE pathogenesis and the subsequent development of PAH (Dhala, 2012). Pro-inflammatory cytokines, particularly Type I Interferons (IFN-α), serve as central mediators by promoting B-cell differentiation and autoantibody production. Within the pulmonary vasculature, cytokines such as IL-6 and TNF-α contribute to chronic vascular inflammation and stimulate the proliferation of pulmonary artery smooth muscle cells (Crow, 2014). Furthermore, elevated TGF-β levels are of particular significance in our proposed model; acting as a potent inducer of EndMT, TGF-β facilitates structural remodeling of pulmonary vessels (Singh et al., 2024). This cytokine milieu establishes a pathogenic feed-forward loop, wherein immune dysregulation directly propels vascular obliteration (Dhala, 2012; Humbert et al., 2023).

To explain these observations mechanistically, we postulate that a principal pathway through which HCQ exerts its protective effect is the inhibition of complement-mediated induction of EndMT. EndMT is a dynamic cellular reprogramming process increasingly recognized as a fundamental driver of PAH pathophysiology. During this transformation, quiescent vascular endothelial cells lose their characteristic lineage markers (e.g., VE-cadherin, CD31) while concurrently acquiring the migratory, proliferative, and matrix-producing properties of mesenchymal cells, characterized by the expression of α-SMA, vimentin, and fibronectin (Ranchoux et al., 2015; Singh et al., 2024). This transdifferentiation directly contributes to the obliterative remodeling of small pulmonary arteries by generating a population of matrix-producing cells within the vascular wall (Ranchoux et al., 2015; Singh et al., 2024).

**FIGURE 2:**
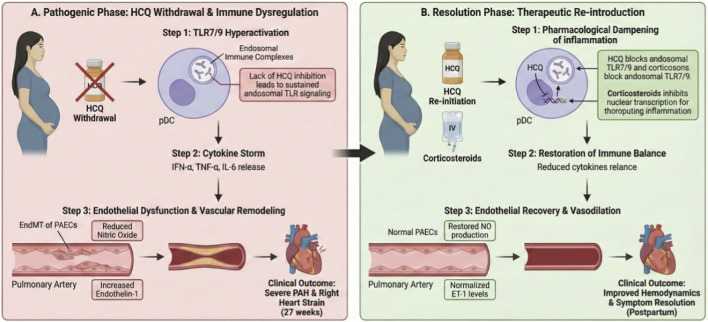
**(A)** The pathogenic cascade following HCQ withdrawal, characterized by the accumulation of immune complexes, endosomal TLR7/9 activation, and subsequent complement dysregulation (C5a generation). This leads to the activation of the C5a-MAPK/ERK axis and drives Endothelial-Mesenchymal Transition (EndMT), resulting in pulmonary vascular remodeling. **(B)** The pharmacological protective mechanism of HCQ. HCQ accumulates in acidic endosomes, elevating endosomal pH and disrupting TLR7/9 activation. This upstream blockade effectively inhibits the complement- EndMT axis, thereby preserving endothelial fidelity and preventing irreversible vascular remodeling.

We propose an integrated mechanistic model (Figure 2) linking SLE activity, complement dysregulation, and the protective action of HCQ. The cascade initiates upon the loss of HCQ-mediated suppression. In this unchecked state, the accumulation of endogenous nucleic acids derived from apoptotic cells and neutrophil extracellular traps facilitates the formation of immune complexes. These complexes act as potent ligands for endosomal Toll-like Receptors (TLRs), primarily TLR7 and TLR9, thereby driving type I interferon production and, crucially, precipitating complement activation (Rainsford et al., 2015; Rainsford et al., 2015). A pivotal consequence is the generation of the anaphylatoxin C5a. Upon binding to its receptor C5aR1, which is abundantly expressed on endothelial cells and leukocytes, C5a initiates potent intracellular signaling. This process prominently involves the MAPK/ERK and NF-κB pathways, which serve as central regulators of cell proliferation, survival, and inflammatory gene expression (Ranchoux et al., 2015; Singh et al.,. 2024).

Sustained activation of the C5a-MAPK/ERK axis serves as a potent driver of EndMT, leading to the transcriptional upregulation of mesenchymal master regulators, including Snail, Twist, and Slug (Ranchoux et al., 2015; Singh et al., 2024). These transcription factors orchestrate the phenotypic switch by repressing endothelial-specific genes while concurrently activating mesenchymal markers (Ranchoux et al., 2015; Singh et al., 2024). Pharmacologically, HCQ functions as a weak base that accumulates within acidic endosomes, thereby elevating endosomal pH. This physicochemical interference disrupts the conformational stability requisite for TLR7/9 activation by nucleic acids, effectively abrogating the upstream trigger of this cascade (Rainsford et al., 2015). By attenuating this initiating signal, HCQ inhibits complement activation and C5a signaling, thereby preventing the transcriptional reprogramming that drives EndMT (Rainsford et al., 2015). Consequently, HCQ acts as an upstream regulatory blockade. This agent preserves endothelial fidelity and precludes the initiation of irreversible vascular remodeling. Such mechanism elucidates the clinical reversibility observed in this case (Singh et al., 2024; Rainsford et al., 2015).

### 3.3 Implications for clinical pharmacology and personalized medicine

The lessons from this case transcend a single patient narrative, offering actionable insights for refining the standard of care. Crucially, we must reframe adherence not merely as behavioral compliance, but as a pharmacodynamic imperative. This case advocates for a fundamental shift in the clinician-patient discourse: HCQ must be presented not as a flexible recommendation, but as an indispensable component of life-saving prenatal care for women with SLE. Counseling must proactively address and dismantle teratogenicity misconceptions with robust safety data. Furthermore, healthcare systems should support this mandate through structured pre-conception clinics, pharmacist-led medication therapy management, and the integration of therapeutic drug monitoring (TDM) to objectively verify adherence and optimize dosing.

Advancing towards biomarker-guided proactive management, the temporal concordance between hypocomplementemia (low C3/C4) and the onset of PAH in this case is compelling. This observation suggests that serial monitoring of complement proteins, alongside traditional markers like anti-dsDNA, may serve as valuable sentinel biomarkers for subclinical vascular risk. A declining complement profile, even in an otherwise clinically stable patient, could act as an early warning signal of impending pathology, prompting intensified surveillance (e.g., scheduled echocardiography) or therapeutic reinforcement before symptomatic, life-threatening PAH develops. Ultimately, this approach embodies the principle of personalized, pre-emptive pharmacotherapy.

By reconceptualizing HCQ from a conventional immunomodulator to a candidate agent with specific efficacy against pulmonary vascular pathology, our hypothesis charts a distinct course for future translational inquiry. First, priority must be given to *in vitro* and animal model studies designed to directly validate capacity of HCQ to inhibit C5a-induced EndMT in pulmonary endothelial cells under SLE-relevant conditions. In parallel, large, prospective pregnancy registries are essential to quantify the magnitude of PAH risk reduction attributable to HCQ and to establish the predictive utility of complement dynamics. Finally, investigation is warranted into the potential additive or synergistic effects between HCQ and approved PAH-targeted therapies (e.g., endothelin receptor antagonists, phosphodiesterase-5 inhibitors) in severe or refractory SLE-PAH phenotypes.

Addressing the potential confounding influence of concurrent corticosteroid therapy is essential to isolate the therapeutic efficacy of HCQ. While corticosteroids remain the cornerstone for mitigating acute systemic flares via broad immunosuppression (Fanouriakis et al., 2023), notably, the patient developed severe PAH despite adherence to low-dose prednisone (5 mg/day), an event temporally coinciding specifically with HCQ cessation. This clinical sequence implies a mechanistic divergence: while low-dose corticosteroids may control systemic lupus activity, they appear insufficient to arrest pulmonary vascular remodeling driven by the Complement-EndMT axis (Kato et al., 2011; Singh et al., 2024). In contrast to the non-specific anti-inflammatory action of steroids, HCQ facilitates lysosomal homeostasis and endothelial stabilization, potentially serving as a non-redundant inhibitor of endothelial phenotypic transition (Schrezenmeier and Dörner, 2020). Therefore, the observed hemodynamic deterioration post-withdrawal and subsequent recovery upon reintroduction substantiate the hypothesis that HCQ exerts distinct pulmonary vasculoprotective effects unreplicated by corticosteroids alone (Kato et al., 2011; Sammaritano et al., 2020).

### 3.4 Limitations

Given the inherent limitations of a single-case study, a definitive causal link between HCQ withdrawal and the specific activation of the Complement-EndMT axis cannot be conclusively established. The concurrent administration of high-dose corticosteroids and vasodilators, while clinically necessary for managing the life-threatening SLE-PAH flare, introduces confounding factors that preclude discerning the independent hemodynamic contribution of HCQ re-initiation.

A notable pharmacological limitation was the absence of therapeutic drug monitoring. Pregnancy associated physiological adaptations including plasma volume expansion and augmented renal clearance significantly alter hydroxychloroquine pharmacokinetics (Pariente et al., 2016). Furthermore, HCQ specific lysosomotropic pharmacodynamics complicate safety interpretation. Acting as a lipophilic weak base the drug sequesters within lysosomes to inhibit antigen presentation and TLR signaling yet carries an inherent risk of pathological accumulation manifesting as cardiomyopathy and retinopathy. Absent serial whole blood quantification precluded precise differentiation between therapeutic lysosomal saturation and toxic tissue loading. Consequently, while the acute clinical deterioration in our patient strongly suggests that the risk-benefit ratio favored HCQ continuation, we lacked the longitudinal pharmacokinetic data to rigorously evaluate the cumulative sequestration burden in this specific maternal-fetal context (Balevic et al., 2019).

Moreover, the proposed mechanism linking complement activation to pulmonary vascular remodeling remains speculative. Given the prohibitive risks associated with invasive procedures during pregnancy, tissue-level validation (e.g., lung biopsy for endothelial phenotyping) and serial hemodynamic monitoring were precluded. Consequently, direct histological evidence of EndMT and quantifiable data on complement deposition in the pulmonary vasculature are lacking.

While we posit that HCQ exerts protective effects via lysosomal stabilization to inhibit the Complement-EndMT axis, this hypothesis warrants validation through future translational research. Prospective longitudinal studies incorporating specific biomarkers (e.g., plasma C3a/C5a levels, endothelial activation markers) and targeted mechanistic *in vitro* experiments are essential to substantiate these molecular pathways and refine precision medicine strategies for SLE-PAH in pregnancy.

## 4 Conclusion

This detailed case report significantly elevates hydroxychloroquine from its established role as a broad-spectrum immunomodulator to that of a pivotal, disease-modifying agent in the prevention of pregnancy-associated SLE-PAH. The clinical narrative provides a stark warning against non-adherence and demonstrates the potential for pharmacological intervention to reverse early-stage disease. The proposed mechanistic model centers on inhibiting the complement-C5a-MAPK/ERK-EndMT axis to bridge autoimmunity and vascular pathobiology. This novel pharmacological perspective offers a robust rationale for observed clinical phenomena. It underscores the necessity of stringent HCQ adherence during SLE pregnancies and suggests that routine, serial complement profiling could refine risk stratification and enable a more proactive, precision-based management approach. Ultimately, protecting the pulmonary vasculature in this high-risk population demands a synergistic strategy comprising intensive patient education, informed and vigilant clinical monitoring, and a renewed recognition of for the multifaceted pharmacology of this cornerstone drug.

## Data Availability

The raw data supporting the conclusions of this article will be made available by the authors, without undue reservation.
